# Gender-based violence among women and girls in conflict-affected areas of Northeast Amhara, Ethiopia

**DOI:** 10.3389/fgwh.2024.1453149

**Published:** 2024-12-18

**Authors:** Endalkachew Dellie, Endalamaw Salelew, Samrawit Mihret Fetene, Wubshet D. Negash, Adane Kebede, Tsegaye G. Haile, Melaku Birhanu Alemu, Jinha Park, Selamawit Tefera, Bruhtesfa Mouhabew Alene, Asmamaw Atnafu

**Affiliations:** ^1^ Department of Health Systems and Policy, Institute of Public Health, College of Medicine and Health Sciences, University of Gondar, Gondar, Ethiopia; ^2^ Department of Psychiatry, School of Medicine, College of Medicine and Health Sciences, University of Gondar, Gondar, Ethiopia; ^3^ Department of Epidemiology for Policy and Practice, National Centre for Epidemiology and Population Health, The Australian National University, Canberra, ACT, Australia; ^4^ Curtin School of Population Health, Curtin University, Perth, WA, Australia; ^5^ Korea Foundation for International Healthcare,Ethiopia Office, Addis Ababa, Ethiopia; ^6^ Institute of Technology, Department of Biomedical Engineering, University of Gondar, Gondar, Ethiopia

**Keywords:** gender-based violence, conflict-affected settings, women and girls, Amhara, Ethiopia

## Abstract

**Background:**

Gender-Based Violence (GBV) is one of the major problems that women and girls encountered during the conflict between the Ethiopian federal government and the Tigray People's Liberation Front (TPLF). However, there is a gap in evidence regarding gender-based violence resulting from the ongoing conflict in these areas. Therefore, this study assessed the prevalence of GBV and its contributing factors in the conflict-affected northeastern Amhara region of Ethiopia.

**Methods:**

A community-based cross-sectional study was conducted from July 7th to September 30th, 2023, among 597 women and girls who lived in the three most conflict-affected districts (Wadla, Lay-Gaynt, and Meket) in northeastern Amhara regional state of Ethiopia. GBV was assessed using the WHO multi-country study on women's health and domestic violence against women questionnaire, which has 13 items and measures three violence domains (emotional, physical, and sexual). A binary logistic regression analysis was conducted. We used Adjusted Odds Ratios (AOR) with their respective 95% Confidence Intervals (CI) and a *p*-value of <0.05 to identify statistically significant factors.

**Results:**

The overall prevalence of gender-based violence was 39.0% (95%CI: 35.2–43.6); 36.7% were experienced emotional, 15.4% physical, and 8.9% sexual violences. The burden is higher among individuals who are divorced, substance users, have low social support, or have participated in the war.

**Conclusions:**

Two-fifths of women and girls in conflict-affected areas of the northeastern Amhara region experienced violence. Thus, collaboration between healthcare providers and policymakers is needed to enhance care for victims, including the provision of social support and substance use mitigation.

## Introduction

Gender-based violence is a global public health problem and one of the major risks that women and girls encounter during situations of armed conflict (1, 2). It is broadly defined as any harmful act that is committed against a person will based on a socially ascribed gender difference and involves various forms of violence such as intimate partner violence (IPV), and other forms like physical, psychological, economic, and sexual violence (3, 4). In conflict settings, the prevalence of GBV rises significantly, with its scale, type, and target populations depending on the context (1, 2, 5). Despite being a common human rights violation, GBV is still not widely recognized, particularly in developing countries (6–8).

The World Health Organization estimates that 27% of women around the globe have experienced some form of violence over the course of their lives (9)*.* However, estimates for the prevalence of sexual violence among women in humanitarian emergencies are quite high, ranging from 21%–53% (10, 11)*.* It has been systematically used as a weapon of war in situations of armed conflict by armed groups and state actors to instill fear and demoralize populations (12, 13). For instance, rape and sexual violence have been used as a tactic of war in Syria as part of government and militant strategies (14).

Since November 2020, Ethiopia has been under armed conflict between the federal government and the Tigray People Liberation Front (TPLF) (15). According to reports from international humanitarian organizations, such as the United Nations and Amnesty International, all parties to the conflict in the Amhara region have committed rape and other forms of sexual violence (16, 17). The reports also show that sexual violence was systematically used as a tool for intimidation and retaliation, which exacerbates the vulnerability of women and girls in these areas (16).

As a consequence of violence, survivors of GBV often suffer several psychological problems, including posttraumatic stress disorder (PTSD), depression, substance abuse, and suicide or suicidal ideation (18, 19). In addition, it increases the risks of HIV and other sexually transmitted infections (STIs), as well as short-and long-term health, economic and social problems for individuals, families, and communities (20). Therefore, GBV is considered to be both a human rights violation in its own right and a major limitation on women's participation equally in political and economic life (21).

Despite the high prevalence of GBV in conflict-affected areas of Ethiopia, there remains a significant gap in support and interventions for survivors. Additionally, there is an evident vacuum in informing policymakers about the problem of gender-based violence victims caused by the ongoing conflict in Ethiopia. Thus, this study aimed to assess both the prevalence of GBV and its associated factors in the conflict-affected areas of Northeastern Amhara.

## Method and materials

### Study design and settings

A community-based survey was conducted from July 7th to September 30th, 2023 in the three most conflict-affected districts (Wadla, Lay-Gaynt, and Meket) in northeastern Amhara regional state, Ethiopia.

In these districts, infrastructure was severely destroyed, looted, and damaged. Numerous crimes were reported, including the deliberate targeting of public services. For example, the Federal Ministry of Health of Ethiopia reported, that over 1,500 healthcare facilities across the Amhara and Afar regions were either destroyed, looted, or purposefully demolished during the conflict. Hence, the aforementioned factors, along with the pressing necessity to understand the magnitude of GBV in conflict-affected areas, influenced the selection of the study site.

Based on the 2007 national census conducted by the Central Statistical Agency of Ethiopia (CSA), the pridicted population in these districts for 2024 was 545,389, of which 277,940 (50.96%) were women (22). Meket and Lay-Gaynt districts accounted for 226,644 and 206,499 populations, respectively. Moreover, Meket and Wadila districts are found in the north Wollo zone, whereas the Lay-Gaynt district is located in the North Gondar Zone, Amhara Regional State. The distances from Bahir Dar (the capital city of Amhara regional state) to these districts are 245, 268, and 177 kilometers to the east, respectively.

### Sample size determination, study population and sampling procedure

The sample was calculated using a single population proportion formula with the assumptions of a proportion of GBV of 50% (to get the maximum sample), a 95% confidence level and a 5% marginal error (d). A design effect of 1.5 was applied to account for potential variability in GBV prevalence between districts, although this variability was assumed to be minimal, as all three districts were similarly affected by the conflict. Adding a 10% non-response to account for potential dropouts, the final sample size was determined to be 605.

Multistage sampling was used to select women and girls over the age of 15 years residing in the three districts included in the study. Initially, all Kebeles within each district were listed and a total of nine kebeles (the lowest administrative unit) were selected using the lottery method, by ensuring proportional representation based on the total number of Kebeles in each district. Specifically, four Kebeles were chosen from Meket district (28 Kebeles), three from Lay-Gaynt district (21 Kebeles), and two from Wadila district (14 Kebeles).

Then, The calculated sample size was allocated proportionally for each selected Kebeles based on the number of women and girls within each kebele. Finally, using the Community Health Information System (CHIS) register as a sampling frame, households (HHs) were selected through simple random sampling techniques (lottery method). Then, interviews of the women and girls were conducted from home to home. If more than one respondent was available in the selected HH, one illegible woman or girl was randomly selected to be interviewed, and if the selected HH didn't have an illegible woman girl age greater than 15 years, the next HH was considered until the target sample size was reached.

### Variables and measurements

The outcome variable of this study was gender-based violence against women. It was assessed using the WHO multi-country study on women's health and domestic violence against women questionnaire, which has 13 items and measures three violence domains (23). Of these four questions about emotional violence, six are about physical violence, and three are about sexual violence. Answering “yes” to any question from 13 items is considered violence against women, and responding “yes” to any question in each domain is verified as physical, emotional, and sexual violence against women (23).

Perceived stigma was assessed using perceived devaluation and discrimination (PDD). The PDD is a 12-item tool that measures on a 4-point Likert scale with possible scores ranging from 1 to 4 (1 = strongly disagree, 2 = disagree, 3 = agree, and 4 = strongly agree). A high level of PDD is indicated by agreement with six of the items and disagreement with six others. Items 1, 2, 3, 4, 8, and 10 were scored in the reverse direction. The prevalence of high perceived stigma was defined as an item mean score of 2.5 or higher on the mean aggregated scale score (this criterion represented the “midpoint” on the 1–4 item scale) on PDD scales. Responses scoring 2.5 or above indicate “high perceived stigma,” while scoring below represents “low perceived stigma” (24).

The patient health questionnaire-9 (PHQ-9) depression screening and the diagnostic tool were used to assess depression level which has nine items. Scores for each item range from 0 (“not at all”) to 3 (“nearly every day”), with a total score ranging from 0 to 27. About the cutoff point (0–4 = No minimal depression, 5–9 = Mild depression, 10–14= Moderate depression, 15–19 = Moderately severe depression, and 20–27 = severe depression), the questionnaire has a sensitivity of 88% and a specificity of 88% for major depression and was validated in Ethiopia (25).

The perceived social support status was assessed using the Multidimensional Scale of Perceived Social Support (MSPSS), which is designed to measure an individual's level of perception of social support from three sources: family, friends, and a significant other. Across many studies, the MSPSS has been shown to have good internal reliability, validity, and a fairly stable factorial structure. It has 12 items, each rated on a 7-point Likert scale ranging from “1” (very strongly disagree) to “7” (very strongly agree), with a total scores ranging from 12 to 84. The mean scale scores are categorized into three levels: a score ranging from 1 to 2.9 considered low support, 3 to 5 as moderate support, and 5.1 to 7 indicates high support. Similarly, the total scores are classified into three ranges, with 12–35 reflecting low perceived social support, 36–60 moderate perceived social support, and 61–84 high perceived social support (26).

Post-traumatic stress disorder was assessed with the post-traumatic stress disorder checklist for the Diagnostic and Statistical Manual for Mental Disorders_5 (PCL-5). The PCL_5 has 20 items on a 5-point Likert scale ranging from 0 = not at all, 1 = a little bit, 2 = moderately, 3 = quite a bit, and 4 = extremely. Items summed to provide a total severity score (range 0–80), and using a total score of 31–33 or higher indicates post-traumatic stress disorder (27).

### Data collection procedures

Data were collected using a structured interviewer-administered questionnaire. The Questionnaire was initially prepared in English, translated into the local language (Amharic), and then translated back into English by a team of experienced professionals, who were fluent in both languages to ensure its consistency and accuracy. Additionally, a review committee of local cultural advisors and public health experts was involved to ensure the questionnaire's cultural relevance and appropriateness for the target population. Fifteen BSc nurse data collectors and eight MPH specialist field supervisors were employed for the data collection process. Two days of training were provided on handling ethical issues, managing distressing situations, interviewing techniques, maintaining privacy and confidentiality before the actual data collection. The tool was pre-tested on 5% of the sample (31 women and girls) in Gondar Zuria district to ensure the internal validity of the study. The internal consistency of the tool was evaluated with Cronbach's alpha and found to be greater than 0.83.

### Data management and analysis

The collected data were downloaded from the Kobo tool with SPSS and analyzed with binary logistic regression analysis. Descriptive variables were explained with frequency, percentage, tables, and graphs. In the bivariable logistic regression analyses, variables with a *p*-value of less than 0.2 were candidates for multivariate logistic regression analyses to make sure potentially significant predictors were not excluded early. This threshold is widely recognized to improve model robustness by taking confounders and interactions into account, even for variables with marginal individual associations. The independent variables that scored a *p*-value of less than 0.05 in multivariable logistic regression analyses were considered statistically significant. The strengths of the association were described with an adjusted odds ratio and a 95% confidence interval. The Hosmer-Lemeshow statistic has been done and revealed a Chi-square value of 6.001 with significance at a *p*-value of 0.647, which means that the model has a good fit.

## Results

### Sociodemographic characteristics of participants

A total of 597 participants were involved in this survey, with a response rate of 98.7%. The mean age of the participants was 30.15 (SD ± 8.63) years. The majority of the participants were married (64.5%), had children (80.1%), identified as Christians (94.8%), had attained secondary-level education (33.3%), were housewives by occupation (35.2%), and lived with their husbands (59.6%) (Table 1).

**Table 1:** Percentage distribution of sociodemographic characteristics among women and girls in conflict-affected areas in the north-eastern Amhara region, Ethiopia, 2023 (*n* = 597).

Variables	Category	Frequency (*N*)	Percent (%)
Age	Mean (±SD)	30.15	8.63
Marital status	Single	120	20.1
	Married	385	64.5
	Divorced	62	10.4
	Widowed	19	3.2
	Separated	11	1.8
Number of children	Has no children	119	19.9
	Number of children 1–3	359	60.1
	Number of childre*n* ≥ 4	119	19.9
Educational status	Not attended formal education	99	16.6
	Primary school education (grades 1–8)	169	28.3
	Secondary school education (grades 9–12)	199	33.3
	College and above	130	21.8
Religion	Cristian	566	94.8
	Muslim	31	5.2
Occupation	Government Employee	80	13.4
	Housewife	210	35.2
	Farmer	100	16.8
	Daily laborer	26	4.4
	Merchant	95	15.9
	Student	66	11.1
	Others^a^	20	3.4
Living conditions	Alone	100	16.8
	With husband	356	59.6
	With family (parents, siblings)	136	22.8
	With Others^b^	5	0.8

^a^ Others include waiters, guards, and house servants.

^b^ Others include living with their madam and living with other families (uncle, aunt).

### Clinical, behavioral and psychosocial conditions of study participants

Among the participants, 7.9% had a history of chronic conditions such as HIV/AIDS, 28.1% reported ever using substances, and 6.7% indicated current substance use. The study also revealed that 55.4% of participants experienced high levels of perceived stigma, 29.7% reported depression, and 25.0% suffered from post-traumatic stress disorder (Table 2).

**Table 2:** Percentage distribution of clinical, behavioral, and psychosocial conditions among women and girls in conflict-affected areas in the north-eastern Amhara region, Ethiopia, 2023 (*n* = 597).

Variables	Category	Frequency (*N*)	Percent (%)
Chronic conditions (HIV/AIDS, DM, HTN)	Yes	47	7.9
	No	550	92.1
History of childhood sexual abuse	Yes	40	6.7
	No	557	93.3
Presence of additional vulnerability	Yes	584	97.8
	No	13	2.2
War participation	Yes	216	36.2
	No	381	63.8
Ever substance use	Yes	168	28.1
	No	429	71.9
Current substance use	Yes	135	27.6
	No	462	77.4
Perceived stigma	Low perceived stigma	266	44.6
	High perceived stigma	331	55.4
Perceived social support	Low social support	29	4.9
	Moderate social support	145	24.3
	High social support	423	70.9
Depression	No minimal depression	414	69.3
	Mild depression	102	17.1
	Moderate depression	31	5.2
	Moderate severe depression	37	6.2
	Severe depression	13	2.2
Post-traumatic stress disorder	Low probability of PTSD	448	75.0
	High PTSD	149	25.0

### Prevalence of gender-based violence

**Figure 1:**
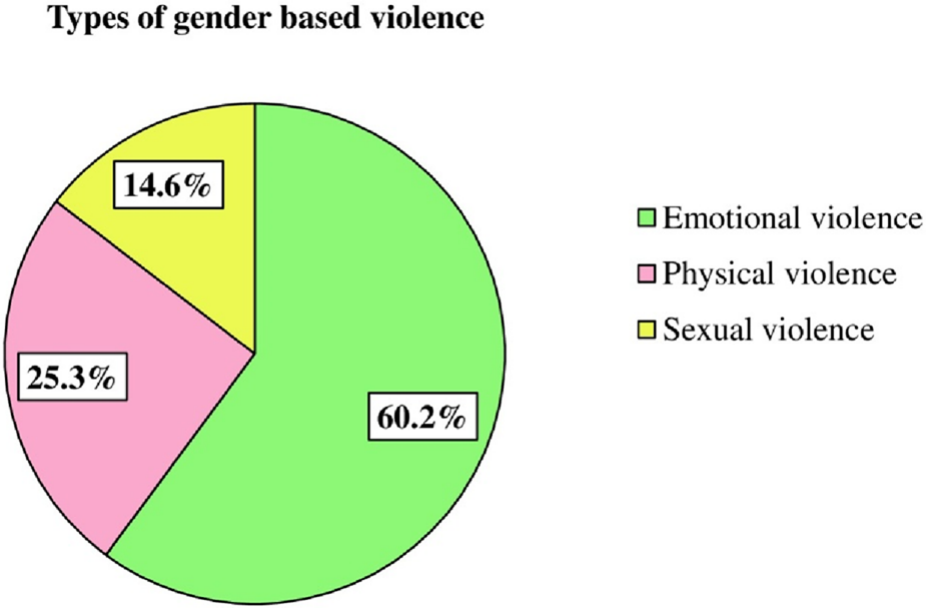
Types of gender-based violence among women and girls in conflict-affected areas in the north-eastern Amhara region, Ethiopia, 2023 (*n* = 597).

This community survey assessed the prevalence of gender-based violence using the World Health Organization's gender-based violence assessment tool. The finding revealed that 39.0% of respondents 95% CI (35.2, 43.6) experienced some form of GBV. Among those affected, 36.7% reported emotional violence, 15.4% reported physical violence and 8.9% experienced sexual violence (Figure 1).

Regarding the pattern of gender-based violence; insulting or making someone feel bad was the most prevalent violence, affecting 27.1% of participants. Conversely, threatened using weapons was the lowest reported form of violence, at 3.0%. Most women experienced different types of violence at different times (Table 3).

**Table 3:** Patterns of gender-based violence using a 13 item wHO GBV screening scale among women and girls in conflict-affected areas in north-eastern Amhara, Ethiopia, 2023 (*n* = 597).

S. no.	Questions	Yes *N* (%)	No *N* (%)
Emotional violence domain			
1.	In the past 12 months, did anyone insult you or make you feel bad about yourself?	162 (27.1)	435 (71.9)
2.	In the past 12 months, did anyone belittle or humiliate you in front of other people?	104 (17.4)	493 (82.6)
3.	In the past 12 months, did anyone do things to scare or intimidate you on purpose (e.g., by the way he looked at you, by yelling and smashing things)?	116 (19.4)	481 (80.6)
4.	In the past 12 months, did anyone threaten you to hurt?	103 (17.3)	494 (82.7)
Physical violence domain			
5.	In the past 12 months, did anyone slap you or throw something at you that could hurt you?	60 (10.1)	537 (89.9)
6.	In the past 12 months, did anyone push you, shove you or pull your hair?	30 (5.0)	567 (95.0)
7.	In the past 12 months, did anyone hit you with his fist or with something else that could hurt you?	42 (7.0)	555 (93.0)
8.	In the past 12 months, did anyone kick you, drag you, or beat you up?	39 (6.5)	558 (93.5)
9.	In the past 12 months, did anyone chok, or burn you on purpose?	23 (3.9)	574 (96.1)
10.	In the past 12 months, has anyone threatened to use or actually used a gun, knife or other weapon against you?	18 (3.0)	579 (97.0)
Sexual violence domain			
11.	In the past 12 months, did anyone force you to do something sexual that you found degrading or humiliating?	31 (5.2)	566 (94.8)
12.	1. In the past 12 months, did anyone physically force you to have sexual intercourse when you did not want to?	2. 37 (6.2)	3. 560 (93.8)
13.	4. In the past 12 months, did anyone have sexual intercourse you did not want to because you were afraid of what the perpetrator might do?	5. 30 (5.0)	6. 567 (95.0)

### Factors associated with GBV

Binary logistic regression was fitted to identify factors that had an association with GBV. Consequently, eight variables with a *p*-value of less than 0.2 during bivariable logistic regression were entered into multivariable logistic regression. Then, five variables namely; marital status, ever substance use, war participation, witnessed maternal violence, and perceived social support, were significantly associated with gender-based violence at *p*-values of less than 0.05.

The odds of GBV among divorced respondents were nearly three times higher than those who were married (AOD): 2.87; 95%CI: 1.19, 6.94). Respondents with low perceived social support and witnessing family violence were at more than three times higher risk of gender-based violence as compared to their counterparts (AOD: 3.10; 95%CI: 1.19, 8.06) and (AOD: 3.27; 95%CI: 1.26, 8.53), respectively.

Those respondents who participated in the war had a nearly two times higher risk of GBV than their counterparts (AOD: 1.78; 95%CI: 1.21, 2.64). The odds of experiencing GBV among respondents who ever used substances were 1.9 times higher than those who did not ever use substances (AOD: 1.90; 95%CI: 1.02, 3.54) (Table 4).

**Table 4:** Bivariate and multivariate logistic regression analysis of factors associated with gender-based violence among study participants, 2023, (*n* = 597).

Variables	Category	GBV			COR (95% CI)	AOR (95% CI)
		No, *n* (%)		Yes, *n* (%)		
Age	Mean (±SD)	30.15 (±8.63)			1.02 (1.00,1.04)	1.01 (0.99,1.04)
Marital status	Married	247 (64.2)	138 (35.8)		1	1
	Single	72 (60.0)	48 (40.0)		1.19 (0.78,1.82)	1.61 (0.82,3.18)
	Divorced	27 (43.5)	35 (56.5)		2.32 (1.35,4.00)	2.87 (1.19,6.94)*
	Widowed/separate	18 (60.0)	12 (40.0)		1.19 (0.56,2.55)	0.64 (0.21,1.95)
Occupation	Government employee	43 (53.8)	37 (46.2)		1.58 (0.94,2.67)	1.66 (0.91, 3.04)
	Private employee	185 (60.3)	122 (39.7)		1.21 (0.84,1.74)	1.10 (0.70,1.74)
	Housewife	136 (64.8)	74 (35.2)		1	1
Living conditions	With husband	228 (64.0)	128 (36.0)		1	1
	Alone	52 (52.0)	48 (48.0)		1.64 (1.05,2.57)	0.94 (0.43,2.07)
	With others*	584 (59.6)	57 (40.4)		1.21, (0.81,1.80)	1.11 (0.60,2.06)
Witnessed maternal violence	Yes	9 (34.6)	17 (65.4)		3.09 (1.21,7.90)	3.10 (1.19,8.06)*
	No	355 (62.2)	216 (37.8)		1	1
War participation	Yes	110 (50.9)	106 (49.1)		1.93 (1.37,2.71)	1.78 (1.21,2.64)**
	No	254 (66.7)	127 (33.3)		1	1
Ever substance use	Yes	97 (57.7)	71 (42.3)		1.21 (0.84, 1.74)	1.90 (1.02, 3.54)*
	No	267 (62.2)	162 (37.8)		1	1
Perceived social support	Low social support	11 (37.9)	18 (62.1)		2.89 (1.33,6.27)	3.27 (1.26,8.53)*
	Moderate social support	83 (57.2)	62 (42.8)		1.32 (0.90,1.94)	1.15 (0.73, 1.80)
	High social support	270(63.8)	153(36.2)		1	1

Significance of association * < 0.05, *** < 0.001, 1 reference, Crude Odds Ratio (COR), Adjusted Odds Ratio (AOR), Confidence interval (CI).

others*: living conditions includes parents, sibling, and living with other families (uncle, aunt).

## Discussion

This study assessed the prevalence of gender-based violence and its contributing factors in conflict-affected settings. The result revealed that significant proportions, 39.0% of the participants had experienced gender-based violence. Our finding was consistent with those of previous works conducted in Ethiopia, 37.9% and 43.3% (28, 29).

However, our study found lower proportions compared to other studies conducted in war-affected areas, such as South Sudan (50%–65%) (30), in a multi-country cross-sectional study (South Kivu, Democratic Republic of Congo, and South Sudan refugees in Ethiopia) (51.62%) (10), Liberia (60%) (31), Yemen (50%) (32), Ukraine (70%) (33), and the Kurdistan region in Iraq (99.7%) (34). The variations in prevalence could be attributed to differences in study populations, measurement tools, study designs, participants' sociocultural norms and conflict intensity. Despite our efforts to ensure data quality and handle ethical issues, participants may underreport instances of violence due to fear of repercussions, social stigma, intimidation, and feelings of shame. Therefore, the sensitivity of the issue may be a contributing factor to the underreporting of the problem.

On the other hand, the 39.0% prevalence of gender-based violence found in this study is higher than studies carried out on Syrian refugees at (31.0%) (35), among internally displaced people in southern Nigeria at (22.2%) (36), in Bougainville, Papua New Guinea (37). The possible explanation for the variation in results might be attributed to the difference in the study contexts and populations. Previous studies in Syria, Nigeria, and Guinea were focused on refugee populations, often within displacement camps where international monitoring and aid might provide some protective measures against GBV. Whereas, the current study is conducted in a community that has survived in politically unsecured moments, potentially increased GBV risks due to limited external support and disrupted local structures.

The findings of this study revealed that being divorced was significantly associated with gender-based violence. This is supported by the experiences and recommendations of nongovernmental organizations (NGOs) working in humanitarian settings, which emphasize the vulnerability of divorced women to GBV in conflict zones (38). This could be due to the fact that divorced wowen in conflict settings may significantly increase their exposure to gender-based violence because of the breakdown of traditionally structured protection and increased social stigma.

The result of the current study shows a significant association between low perceived social support and gender-based violence. This is supported by reports on gender-based violence in humanitarian settings (28, 39). This might be explained by the disruption of social connections during the conflict. Additionally, the deep-rooted belief in male dominance exposes women to gender-based violence, particularly in times of humanitarian crises when social networks and basic protection mechanisms are interrupted or absent. This suggests that in order to reduce the risks of GBV, social support networks such as peer support groups and family networks need to be strengthened.

The study's findings indicate a significant association between ever substance use and gender-based violence. This result is in line with prior studies carried out in different contexts and periods (40–43). This association may be explained by the fact that psychoactive substances could compromise the decision-making capabilities of both the perpetrator and the victim, thereby increasing the likelihood of violence. Furthermore, many studies showed that having a history of substance use puts them at increased risk of gender-based violence and vice versa (40–43). This implies that preventive actions, such as awareness campaigns about the risks of substance use in relation to GBV, and integrating GBV prevention into substance use rehabilitation programs are essential during conflicts. However, further research is necessary to comprehend the cause-and-effect relationship between substance use and gender-based violence.

This study showed a statistically significant association between participation in war and the incidence of gender-based violence. This finding is consistent with the United Nations High Commissioner for Refugees (UNHCR) report: in times of crisis and during displacement, the threat of GBV significantly increases for women and girls (44). The possible justification could be due to the breakdown of law and order and the destabilizing effects of war, which create an environment that is conducive to GBV. This tactic demoralizes the individuals directly affected as well as the community at large by instilling fear and asserting power. To minimize such risks, policymakers and humanitarian organizations need to take preventive measures, such as establishing secure spaces for women and girls and promoting awareness campaigns during times of war.

Furthermore, the findings of this study reported that women and girls who witness family violence are more likely to experience various forms of gender-based violence. This indicates that witnessing violence within the family can increase vulnerability to different types of violence throughout the lifespan. Therefore, it is important to provide counseling for families exposed to violence, which might help break the cycle of trauma and reduce GBV risk.

### Contributions and limitations of the study

This study is not free from limitations. One of the potential sources of bias is response bias, as it can be challenging to extract information on violence against women where participants may be hesitant to disclose their experiences of violence due to fear of stigma or retribution. This could result in underreporting of violence against women. To minimize this issue, well-trained data collectors who understand the sensitive nature of the topic were employed. They wear gowns, apply culturally sensitive approaches and trauma-informed interviewing techniques.

Another limitation is some households may have relocated due to conflict, which could influence the representation of displaced populations. Additionally, this study was not triangulated with a qualitative method. Despite the above limitations, this study serves as a valuable starting point for addressing the issue of GBV and can inform policy and intervention efforts aimed at reducing violence against women and girls.

## Conclusion and recommendations

In conclusion, the prevalence of GBV in the study area was found to be high as compared to the global average. Therefore, healthcare managers and policymakers need to understand these dynamics, and promote social support interventions, and ensure accountability for perpetrators of GBV in conflict-affected areas. The result also indicated that those who have low social support, substance use, war participation, witnessed family and divorce need governmental and nongovernmental organizations collaborative screening and intervention. Furthermore, further research using a mixed-methods approach could help deepen our understanding of the contextual factors and lived experiences of those affected by GBV.

## Data Availability

The original contributions presented in the study are included in the article/Supplementary Material, further inquiries can be directed to the corresponding author.
